# Case report: Portal vein ligation: lessons from patients with PRETEXT III hepatoblastoma in restoring future liver remnant before major hepatectomy and literature review

**DOI:** 10.3389/fsurg.2023.1152901

**Published:** 2023-06-19

**Authors:** Suiin Gang, Suhyeon Ha, Hyunhee Kwon, Jueun Park, Jung-Man Namgoong

**Affiliations:** Department of Pediatric Surgery, Asan Medical Center, University of Ulsan College of Medicine, Seoul, Republic of Korea

**Keywords:** hepatoblastoma, embolization, portal vein, hepatic failure, liver volume, remnant liver

## Abstract

**Background:**

We describe three cases involving three patients with PRETEXT III hepatoblastoma invading the hepatic hilum. After portal vein embolization, the patients underwent uncomplicated trisectionectomy.

**Methods:**

Medical records between March 2016 and March 2021 were reviewed, and three patients were selected. A literature review of techniques for increasing future liver remnant in children diagnosed with hepatoblastoma was also conducted.

**Results:**

All tumors involved the right lobe and hepatic hilum (PRETEXT III). After neoadjuvant chemotherapy, the tumor size decreased, but hilar involvement was unimproved. Right portal vein ligation (RPVL) was performed to increase the left lobe volume. Post-ligation, the remnant liver increased. Liver function was restored to normal levels within 5 days after the hepatectomy. All patients underwent two cycles of adjuvant chemotherapy without tumor recurrence.

**Conclusions:**

RPVL can be safely performed before extended hepatic resection in children with a giant hepatoblastoma invading the hepatic hilum. The tumor was completely resected by securing a sufficient margin and increasing the residual liver volume with portal vein embolization. The patients recovered and underwent adjuvant chemotherapy without the deterioration of liver function.

## 1. Introduction

Hepatoblastomas (HBLs) account for 80% of pediatric primary liver tumors and occur in children before the age of 5 years (1, 2). The basis of treatment is surgical resection, chemotherapy, and liver transplantation. This combination of treatments has raised the 5-year survival rate to 80% (3). Surgical resection is the definitive treatment, and complete resection with a proper resection margin is essential for a better prognosis. However, it is important to leave a sufficient future liver remnant (FLR) (4). This is to improve the prognosis and prevent progression to hepatic failure after hepatectomy and safely facilitate the progress and completion of adjuvant chemotherapy without deterioration of hepatic function. Therefore, efforts to preserve the remnant liver as much as possible are essential, especially in patients with large tumors.

Portal vein embolization (PVE) has been performed in hepatocellular carcinoma patients to increase the FLR. This has been improved to associated liver partition and portal vein ligation (ALPPS) in recent years (5). However, few PVE cases have been attempted in the pediatric population as several difficulties exist relating to embolization (6). Challenges associated with the procedure and concerns about potential side effects have prevented the expansion of PVE application. Several reports have recently shown that ALPPS effectively augments FLR and leads to successful hepatectomy (7, 8). However, complications that may occur during liver partition, such as bleeding, disruption of Glisson's capsule, adhesion, and problems related to tumor spreading, have not yet been evaluated. Recovery from damage occurring during the dissection and partition of liver parenchyma takes time, and the fact that adjuvant chemotherapy is postponed during that time can also be considered a disadvantage. Regarding tumor spread, a case of tumor recurrence in a child who underwent APLLS has been reported (9).

We performed right portal vein ligation (RPVL) in three patients with a PRETEXT III HBL invading the hepatic hilum. Only portal vein ligation was performed to avoid the risk associated with partitioning. By securing the FLR, it was possible to safely perform hepatectomy in patients likely to undergo liver transplantation. As these patients completed adjuvant chemotherapy without compromising hepatic function, in this study, we introduced portal vein ligation (PVL) as a safe and effective technique for patients with advanced HBLs.

## 2. Materials and methods

We retrospectively assessed the medical records of patients aged < 18 years who underwent hepatectomy for hepatoblastoma between March 2016 and March 2021 in the Department of Pediatric Surgery, Asan Medical Center, and three patients who underwent PVL were identified (IRB No.: 2022-1296). Information on initial oncologic conditions, surgical management, and clinical outcomes during follow-up were collected. The clinical outcomes evaluated were liver function and recurrence-free survival.

### 2.1. Operative technique

**Figure 1:**
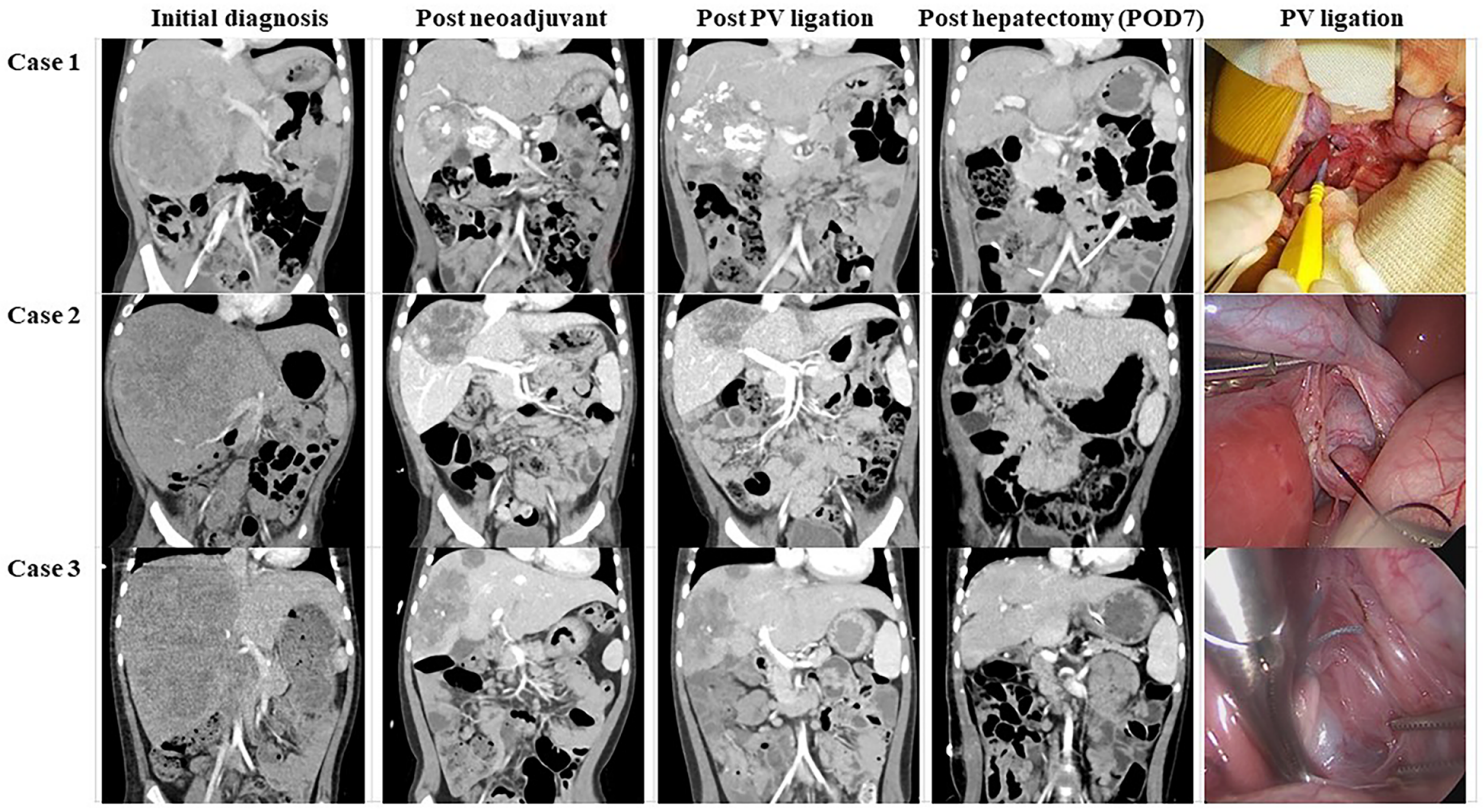
Ct images and intraoperative findings of each patient. The coronal image was used to compare degrees of tumor and liver parenchymal growth. Moreover, intraoperative images revealed that PV ligation was performed without massive dissection or bleeding. *CT, computed tomography; POD, postoperative day(s); PV, portal vein.

The hepatic hilum was dissected, and the right and left portal vein bifurcation was exposed. After identifying and saving the left portal vein, the right portal vein (RPV) was ligated with a nonabsorbable suture. The hepatic artery was saved. RPVL was performed using a laparoscopic approach in two patients and with open laparotomy in the third (Figure 1).

### 2.2. Review of literature

For the literature review, we searched publications from the National Institute of Health database (PubMed.gov) and Google Scholar. The keywords used were “liver partition and portal vein ligation,” “portal vein ligation,” “portal vein embolization,” “future liver remnant,” “hepatoblastoma,” “pediatrics,” and “children.” We reviewed all selected articles in the English language without exclusion due to the paucity of publications.

## 3. Results

**Figure 2:**
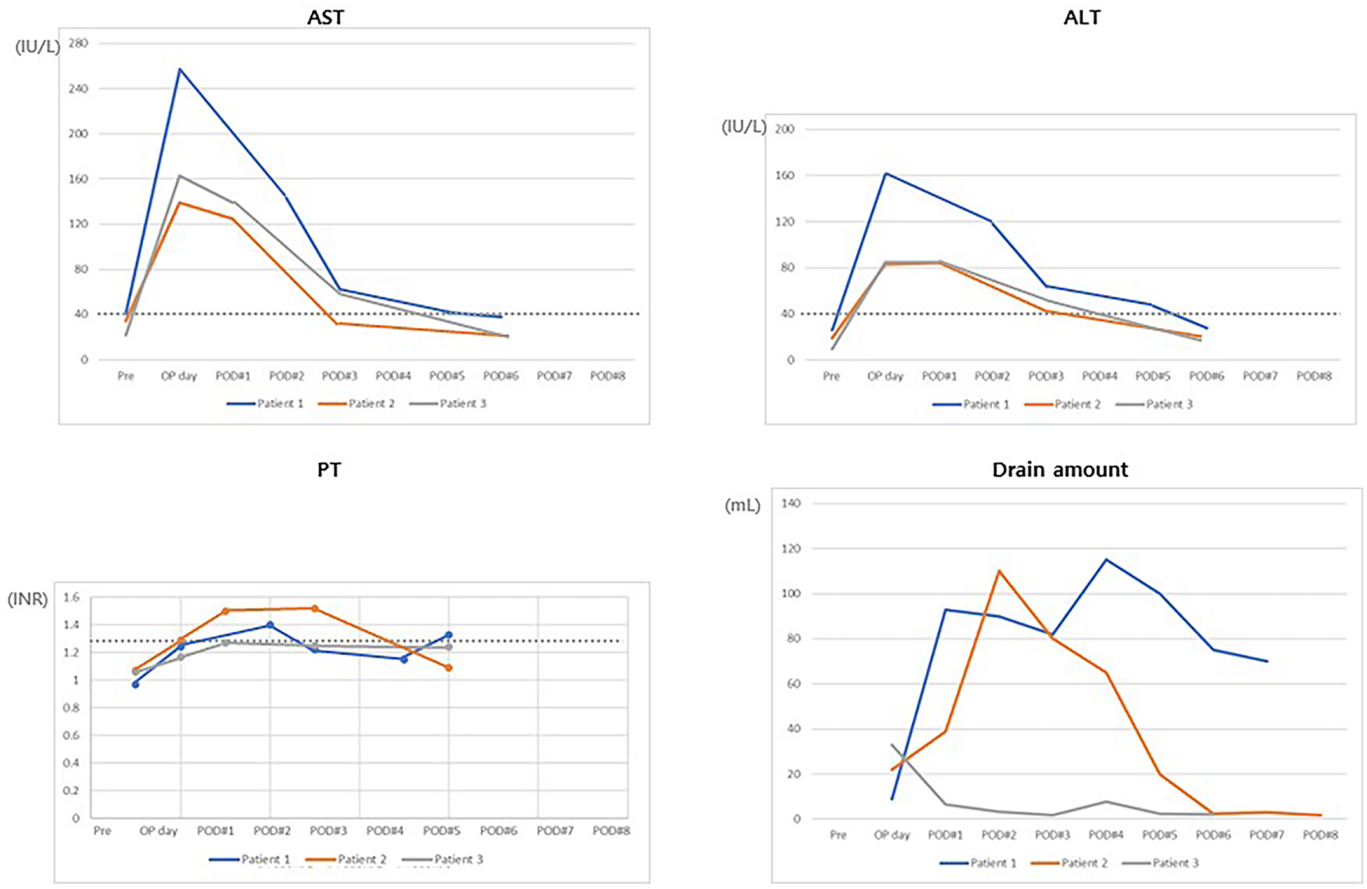
Clinical course after trisectionectomy of the liver. Most parameters returned to normal levels within POD 5. PT was not much different from the normal value. Moreover, all drains were removed before POD 8. AST, aspartate aminotransferase; ALT, alanine aminotransferase; PT, prothrombin time; POD, postoperative day(s).

All patients had PRETEXT III HBLs in three consecutive sections of the right liver (Table 1). Neoadjuvant chemotherapy was commenced with cisplatin, vincristine, doxorubicin, dexrazoxane, and 5-fluorouracil. Subsequently, the tumor size decreased. However, hilar invasion persisted in all three patients, and the tumor extent remained POST-TEXT III. To increase the FLR, we decided to conduct RPVL. All three patients recovered from the operation within a week and underwent trisectionectomy of the liver. Serum liver enzyme values were normalized within 5 ± 1.7 days, and the patients recovered from hepatectomy without complications (Figure 2). They received a single cycle of adjuvant chemotherapy and have been without tumor recurrence until the date of publication of this article. In all three patients, computed tomography (CT) was used to measure the tumor and remnant liver volume (Table 2). A detailed description of each patient is provided below.

**Table 1:** Demographic and basic medical profile of 3 patients with PRETEXT III hepatoblastoma.

Case	Sex	Age at diagnosis (months)	BMI (kg/m^2^)	Tumor size (mm)		PRETEXT staging	Chemotherapy	Operation	AFP (ng/ml)		Histology	Follow-up (days)
				Initial diagnosis	After chemotherapy		Regimen (cycle)		Initial diagnosis	After chemotherapy		
**1**	M	18	16.4	110*90*90	61*58*62	III (V-P-E-F-R-C-N-M-)	POG9645 (5/1)	Rt.trisectionectomy	160,000	6840	Mixed epithelial and mesenchymal type	116
**2**	M	7	15.7	101*100*99	65*63*59	III (V + P + E-F-R-C-N-M+)	POG9645 (5/1)	Rt.trisectionectomy	235,140	769.4	Mixed epithelial and mesenchymal type	1,065
**3**	M	34	14.3	150*85*170	73*45*68	III (V + P + E-F-R-C-N-M+)	POG9645 (4/1)	Rt.trisectionectomy	998,000	231	Mixed epithelial and mesenchymal type	316

PRETEXT, pre-treatment extent of disease; POG9645, cisplatin + vincristine + doxorubicin + dexrazoxane + 5-fluorouracil; AFP, alpha-feto protein.

**Table 2:** Tumor and remnant liver volume measurement of 3 patients with PRETEXT III hepatoblastoma undergoing hepatectomy.

Case	Tumor volume (ml)			Remnant liver volume (ml)			
	Initial diagnosis	After neoadjuvant chemotherapy	After portal vein ligation	Initial diagnosis	After neoadjuvant chemotherapy	After portal vein ligation	After hepatectomy (CT in POD7)
**1**	506	114	104	74	139 (32.6%)	147 (37.6%)	266
**2**	664	63	33	105	87 (32.8%)	122 (44.2%)	204
**3**	1219	333	107	215	212 (39.2%)	286 (56.5%)	334

CT, computed tomogram; POD, post-operative days.

### 3.1. Case 1

An 8-month-old boy without any history of underlying disease visited the hospital for assessment of a painless palpable mass. Under suspicion of an HBL, he was transferred to the pediatric oncology department at Asan Medical Center. Initial serum alpha-fetoprotein and PIVKA II (proteins induced by vitamin K absence or antagonist-II) levels were 1600,000 ng/ml and 977 mAU/ml, respectively. Hepatitis serology revealed that he was free of hepatitis A, B, or C. After a work-up, including a core needle biopsy, an HBL (epithelial type fetal subtype, PRETEXT stage III [V- P- E- F- R- C- N-]) was diagnosed. Tumors were located in S4–S6. Neoadjuvant chemotherapy reduced the size of the tumor and preserve operability. After neoadjuvant chemotherapy, the size decreased from 11.0 × 9.0 × 9.0 cm to 6.6 to 6.4 × 5.7 × 5.9 cm, but the hilar involvement was not resolved. Extrinsic compression of the left and right portal veins was observed on CT. As the patient required trisectionectomy, we planned RPVL to allow for safe hepatectomy. We decided not to perform partitioning due to the risk of tumor seeding. Surgery-related recovery was uncomplicated, and the patient was discharged on postoperative day (POD) 7. No compromise of hepatic function after RPVL was observed. Thirty-seven days after RPVL, a trisectionectomy was performed as the FLR had increased from 139 ml (32.6%) to 147 ml (37.6%). Liver function recovered, and fluid drainage was reduced to less than 20 ml on POD 5. FLR was increased to 266 ml on CT performed on POD 7. He was transferred to the oncology department and finished one cycle of adjuvant chemotherapy. We observed no compromise of liver function during adjuvant chemotherapy. The patient was observed as being well and without recurrence for 116 days.

### 3.2. Case 2

The patient, a 7-month-old boy with a history of abdominal distension, was diagnosed with PRETEXT III (V + P + E-F-R-C-N-M+) HBL. The tumor size decreased from 10.1 × 10.0 × 9.9 cm to 6.6 to 6.5 × 6.3 × 5.9 cm. However, preoperative CT showed tumor involvement of the entire right lobe and S4 and abutment to the RPV and middle and right hepatic veins. Trisectionectomy was indicated, but the measured FLR was 87 ml (32.8%), which was not enough for undergoing hepatectomy and adjuvant chemotherapy. In this patient, liver transplantation was considered a second treatment option to evade postoperative hepatic failure. RPV was selected for the same reasons noted in *Case 1*. The patient was discharged on POD 6 without any surgical complications. Trisectionectomy was performed 45 days after RPVL with sufficient resection margins. He was discharged on POD 8. He was observed as being recurrence-free for 1,065 days.

### 3.3. Case 3

The patient, a 34-month-old boy with a history of fever and abdominal distention, was diagnosed with a PRETEXT III (V + P + E-F-R-C-N-M+) HBL. The initial level of serum alpha-fetoprotein was 998,000 ng/ml. Neoadjuvant chemotherapy was initiated. A 15.0 × 8.5 × 17.0 cm-sized main mass and several discrete daughter nodules in the S4 and right aspect of the liver were identified on CT. After chemotherapy, the tumor size decreased to 7.3 × 4.5 × 6.8 cm, but scattered daughter nodules (up to 3.3 cm in diameter) and attachment to the diaphragm were still observed. RPVL was performed after three cycles of neoadjuvant chemotherapy. The patient was transferred to the oncology department on POD 4. The fourth cycle of chemotherapy proceeded on POD 5 and was finished without complications. Trisectionectomy was performed 52 days after RPVL. After hepatectomy, he was transferred to the oncology department on POD 22 due to a wound problem to complete the adjuvant chemotherapy. The liver function returned to normal range on POD 5. He was observed as being tumor-free for 316 days.

## 4. Discussion

Techniques for overcoming insufficient FLR and succeeding in major hepatectomy have been developed in adult hepatic surgery (4). PVE and ALPPS have expanded the resectability of hepatic tumors. However, these techniques have not gained popularity in the pediatric population because extensive hepatectomy occurs less often, and preoperative hepatic function is usually preserved (6). Moreover, liver transplantation is now the standard treatment in pediatric patients with extensive hepatic tumors. For this reason, there have been a few efforts to perform extended hepatectomy while preserving FLR. Moreover, the FLR threshold is not clearly defined in the pediatric population, and a threshold of 30% in adults with noncirrhotic liver disease is permitted (2).

Although rare, some patients are expected to be cured after hepatectomy because there is no evidence of metastasis despite a large tumor. Traditionally, liver transplantation was the only option for those patients. PVE and PVL to increase FLR and evade post-hepatectomy liver failure have been performed in only a few patients. Since 2014, ALPPS has been introduced and actively adopted for patients with various conditions (7, 10). The mechanical ligation of the portal vein promotes necrosis of the ipsilateral hepatic lobe and hypertrophy of the contralateral lobe by producing growth factors and redirecting portal flow (5). Consequently, FLR increases, and the demarcation between a healthy liver and tumor becomes more prominent.

In a meta-analysis by Fuchs et al., eight cases of PVE, one case of PVL, and 12 cases of ALPPS were reviewed (4, 6, 7). A small-caliber portal vein and scarce experience with the embolic materials used in the adult population limit the expansion of PVE application to children. The use of coil is limited in small children. Therefore, in previous reports, the agent most commonly used for PVE was polyvinyl ethanol (11, 12). Nevertheless, the risk of portal vein rupture and the spread of embolic material to the contralateral portal vein during the procedure remains in small children. Wildhaber et al. reported successful PVE in a 7-month-old patient weighing 6.9 kg, but most experience with PVE is limited to children older than 14 months (12). Thus, PVE seems to be more suitable for older patients. The only report of PVL conducted in children is of a 13-year-old boy with ruptured hepatocellular carcinoma (13). Our experience has demonstrated that PVL safely increased FLR in younger children. Another potential risk regarding PVE is related to continuous heparin infusion and the use of a significant amount of contrast media. Renal function develops until 2–3 years of age, and younger the child, the higher the possibility of complications related to immature renal function. When heparin and contrast media are retained in the body, the bleeding tendency may increase, and acute renal injury may occur due to contrast media. Treatments such as massive hydration may be helpful, but such management in children is not as easy as in adults. These may act as a hurdle to extending the application of PVE to broader age groups. Additionally, damage to the contralateral portal vein by the guidewire during the procedure and the subsequent stricture can be considered other possible complications during PVE.

For PVE, hospitalization for 2–4 days after the procedure was required for follow-up (12). Hospitalization required for PVL was six days for the open approach and 5 days for the laparoscopic approach. Chemotherapy could be resumed within 1 week after surgery. This is comparable to PVE. Furthermore, considering that PVE should be performed under general anesthesia in pediatric patients, the risk related to general anesthesia is similar to that of PVL. Therefore, we concluded that PVL could be considered equivalent, even safer, to PVE in pediatric patients of a broader age group.

The ALPPS performance seems excellent considering the FLR increase rate per unit time (6, 8, 10). In most reports, enhancement of FLR up to 80% was achieved within 6 days. Although ALPPS is attractive in terms of performance, it is an invasive procedure. The mortality associated with ALPPS is 16%–64% and is caused by bile leakage and sepsis (14). Moreover, mortality is as high as 12%–23%. Bleeding during partitioning may delay recovery from the first stage of surgery. Although it was a single case, early multifocal recurrence of HBL after ALPPS was reported by Qazi et al. in a (9). Partitioning disrupts the surgical field, hindering the identification of surgical margins during the second operation. Partitioning of the liver parenchyma acts as a dissemination pathway when lymphovascular or perineural invasion around the tumor is disrupted. Disruption of Glisson's capsule during partitioning may accelerate tumor spread. In this regard, long-term outcome confirmation is essential when permanently introducing ALPPS in children.

We performed ligation without partitioning to avoid the risk of bleeding, tumor spread, and disruption of Glisson's capsule. In addition, we aimed for rapid recovery allowing adjuvant chemotherapy to proceed during the postoperative 4–6 weeks required to increase FLR. The laparoscopic approach shortens hospital stays and may improve the outcome of PVL. As interventional radiology develops, PVE has demonstrated applicability in the pediatric population. However, we demonstrated that PVL can be performed on par with PVE and can augment FLR with lower risk than PVE. In addition, we showed that FLR continued to increase after hepatectomy, and liver function recovered rapidly within 5 days after hepatectomy. This demonstrates the short-term effect of PVE.

Liver transplantation (LT) may be considered as an option for these patients. Recent advances in LT outcomes for hepatoblastoma are encouraging, but when considering salvage LT as a treatment option, primary hepatectomy remains the more reasonable choice due to potential complications associated with transplantation. One such complication is *de novo* cancer and post-transplant lymphoproliferative disorder (PTLD), both of which can pose a significant threat to patient survival.

However, there are some limitations to our study. As this was a small retrospective study with three patients and short-term outcomes, large-scale studies and the evaluation of long-term effects are necessary. Well-controlled comparative studies with ALPPS and PVE are also needed. Finally, the evaluation of the long-term efficacy and safety of this technique will be completed after the comparison of long-term outcomes with liver transplantation.

## 5. Conclusion

Our result indicates that PVL is a safe way to secure FLR, comparing the potential risks of PVE and ALPPS. It guarantees complete resection with clear margins and prevents hepatic failure after hepatectomy in patients with advanced HBL.

## Data Availability

The raw data supporting the conclusions of this article will be made available by the authors, without undue reservation.
